# MicroRNA-Related Strategies to Improve Cardiac Function in Heart Failure

**DOI:** 10.3389/fcvm.2021.773083

**Published:** 2021-11-19

**Authors:** Huatao Zhou, Weijie Tang, Jinfu Yang, Jun Peng, Jianjun Guo, Chengming Fan

**Affiliations:** ^1^Department of Cardiovascular Surgery, The Second Xiangya Hospital, Central South University, Changsha, China; ^2^Department of Pharmacology, Hunan Provincial Key Laboratory of Cardiovascular Research, Xiangya School of Pharmaceutical Sciences, Central South University, Changsha, China; ^3^Hunan Fangsheng Pharmaceutical Co., Ltd. Changsha, China

**Keywords:** heart failure, non-coding RNA, microRNA, modification, therapy

## Abstract

Heart failure (HF) describes a group of manifestations caused by the failure of heart function as a pump that supports blood flow through the body. MicroRNAs (miRNAs), as one type of non-coding RNA molecule, have crucial roles in the etiology of HF. Accordingly, miRNAs related to HF may represent potential novel therapeutic targets. In this review, we first discuss the different roles of miRNAs in the development and diseases of the heart. We then outline commonly used miRNA chemical modifications and delivery systems. Further, we summarize the opportunities and challenges for HF-related miRNA therapeutics targets, and discuss the first clinical trial of an antisense drug (CDR132L) in patients with HF. Finally, we outline current and future challenges and potential new directions for miRNA-based therapeutics for HF.

## Introduction

Heart failure (HF) is a complex clinical syndrome with varied pathophysiology that occurs because cardiac output is insufficient to meet the metabolic needs of organs and tissues in the body (1). HF is very common, with an estimated prevalence of 1–3% in the adult population worldwide, and its incidence rises with advancing age (2). Despite substantial therapeutic advances over the past decades, HF remains a major cause of morbidity and mortality worldwide (1). HF survival rates are no better than they were a decade ago, with an ~53% 5-year survival rate from diagnosis (3). Hence, HF is a global public health problem that requires urgent attention to open a window of opportunity for early diagnosis/prognosis, prevention, and treatment (4).

MicroRNAs (miRNAs) are natural, endogenous non-coding single-stranded RNA molecules of around 22 nucleotides that regulate the expression levels of various genes through Watson-Crick base pairing with target messenger RNAs (mRNAs) at the posttranscriptional level, via binding to the 3′ untranslated regions (UTRs) of target mRNAs (5). The role of miRNAs was first described in *Caenorhabditis elegans*, where silencing of the *lin-14* mRNA at various time points during growth resulted in the normal development of a worm from an embryo (6). During the last few decades, increasing numbers of investigations have indicated the regulatory functions of miRNAs in a diverse range of cellular biological processes (7). According to the latest release of miRBase, more than 2500 miRNAs have been reported to exist in the human genome to date (8). Furthermore, bioinformatic analyses have indicated that the expression levels of 30% of human protein-coding genes are regulated by miRNAs through a series of complex signaling pathways (9).

Encyclopedia of DNA Elements (ENCODE) is an international cooperative project that aims to establish a comprehensive database for human genome data research by integrating DNA, RNA, protein, epigenetic modification, and other levels of data (10–12). The ENCODE project is another tremendous achievement of the international scientific community in the field of genomics, following the Human Genome Project, and is an approach that facilitates improved understanding of miRNAs and their effects in disease pathogenesis (13, 14).

In this review, we give an overview of different types of chemical modifications and viral-vector-based delivery systems used for miRNA modulation in HF, as well as summarizing the opportunities and challenges for miRNAs as therapeutic targets for the treatment of HF. Furthermore, we discuss CDR132L, the first miRNA drug used to treat HF, and future prospects for such therapies.

## MiRNA Biogenesis and Functions

**Figure 1:**
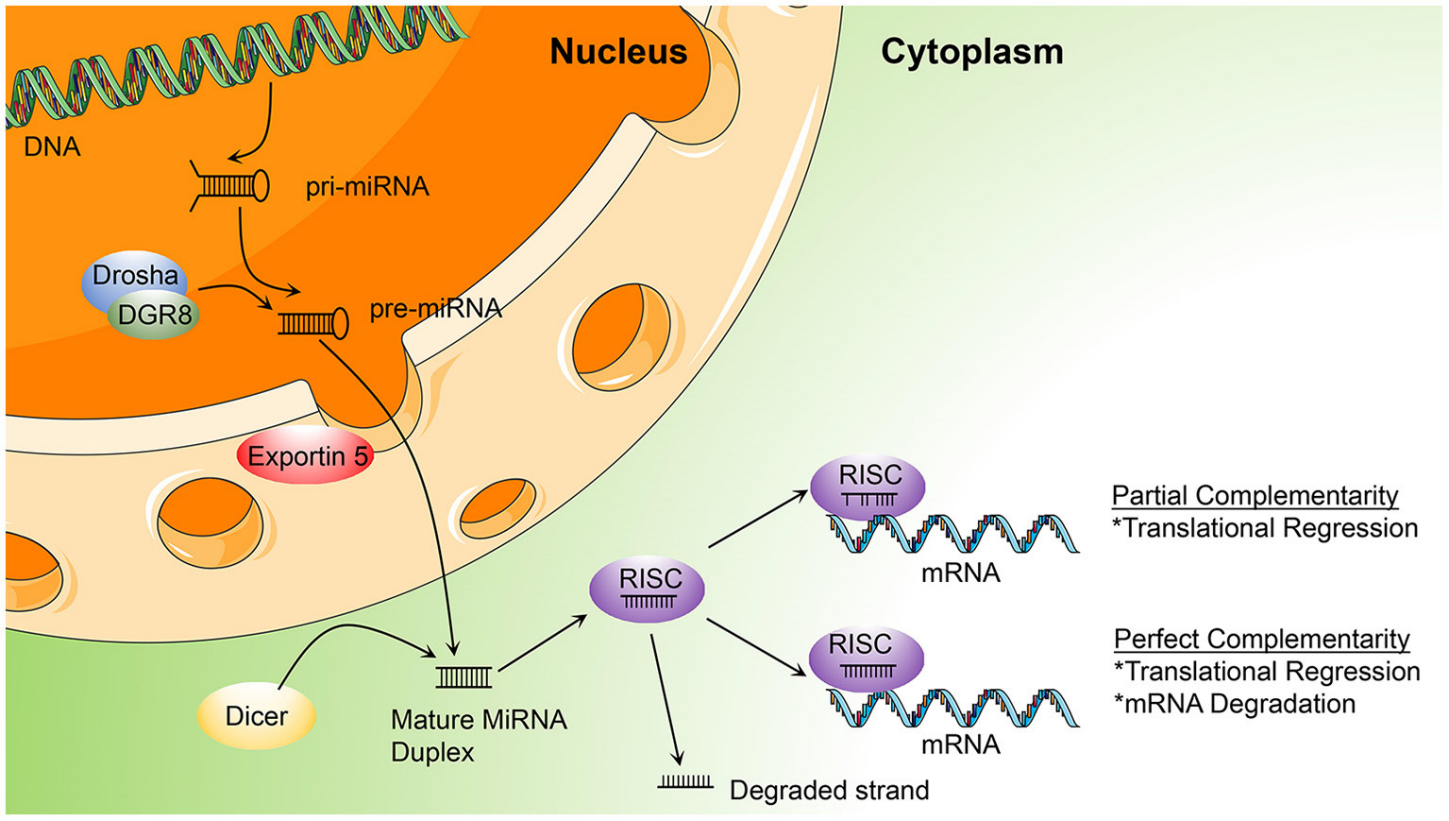
Illustration of the process of microRNA (miRNA) biogenesis and function, described in miRNA biogenesis and functions (DGCR8, DiGeorge syndrome critical region gene 8; RISC, RNA-induced silencing complex; pri-miRNA, primary miRNA; pre-miRNA, precursor microRNA).

The synthesis of miRNA begins with the transcription of miRNA genes by RNA polymerase II. This transcriptional process, which involves transcription factors in a similar way to protein-coding transcripts, leads to the generation of A-tail-capped long transcripts, referred to as primary microRNA or pri-microRNA (15, 16). In the nucleus, a microprocess complex containing two important constituents, the double-stranded RNase III enzyme, DROSHA, and the cofactor, DiGeorge syndrome critical region 8 (DGCR8), processes pri-miRNA to generate precursor miRNA or pre-miRNA (17, 18). In the cytoplasm, pre-microRNAs are further cleaved by the cytoplasmic nuclease, DICER, to produce double-stranded RNA molecules of 18–22 nucleotides, cut from the stem of the hairpin, similar to the double-stranded structure of small interfering RNA (siRNA) (19). The duplex comprises a guide strand (mature miRNA) and a passenger strand (miRNA^*^) (20). While loading on RISC, only the mature miRNA will become active in the silencing procedure, while the miRNA^*^ is usually non-functional and is degraded (21). A multi-protein RNA-silencing complex composed of RISC and miRNA (22) recognizes target RNA molecules by Watson-Crick base pairing to partially complementary sites, mainly located in the 3′ UTR of target mRNAs, and can inhibit the function of corresponding genes in several ways, as follows: blocking initiation and elongation, forcing premature termination of translation, deadenylation of mRNAs to prevent their reuse, and (most importantly) mRNA degradation (23–27) (Figure 1).

## MiRNA Functions in Cardiovascular System Development

The heart is the first organ to form and function during embryonic development. For normal morphogenesis and function, the development process must be uninterrupted (28). MiRNAs can manipulate cardiac gene expression at the posttranscriptional level (29). The requirement for miRNAs in cardiovascular development and function was initially proven by the deletion of the tissue-specific gene, *Dicer1*, in mice*; Dicer1* encodes an enzyme essential for miRNA processing (30), and its deletion in vascular lineages and myocardial tissue results in embryonic lethality and defective heart morphogenesis (30, 31). Subsequently, numerous miRNAs that contribute to heart morphogenesis have been identified; for example, *miR-1* and *miR-133* contribute to cardiomyocyte proliferation, as well as the *miR-15* family, thereby regulating congenital heart disease development or regeneration (32–35).

Several myomirs, including *miR-1, miR-133a, miR-208a, miR-208b*, and *miR-499*, play important roles in normal embryological development of the heart and precise regulation of these myomirs is crucial for normal cardiac development (36). The field of cellular reprogramming and trans-differentiation is rapidly evolving, and cardiac fibroblasts have been demonstrated to directly differentiate into induced cardiomyocytes (iCMs) under the combined influence of the transcription factors GATA4, MEF2C, and TBX5 (GMT) (37). Subsequently, Jayawardena et al. (38) demonstrated that the combination of *miR-1, miR-133, miR-208*, and *miR-499* also function to directly convert fibroblasts to a cardiomyocyte-like phenotype *in vitro*. Among these miRNAs, *miR-133* is considered to mediate the further maturation of transdifferentiated iCMs (39). The importance of miRNA-mediated post-transcriptional regulation in cardiovascular homeostasis, as well as its impact on the pathogenesis, diagnosis, and prognosis of heart disease, is established in the scientific community. Understanding the mechanisms underlying heart development may provide new perspectives relevant to cardiac reprogramming technology, which could, in turn, pave the way for development of miRNA-based approaches for heart disease therapy in the future.

## Chemical Modifications of MiRNAs

**Figure 2:**
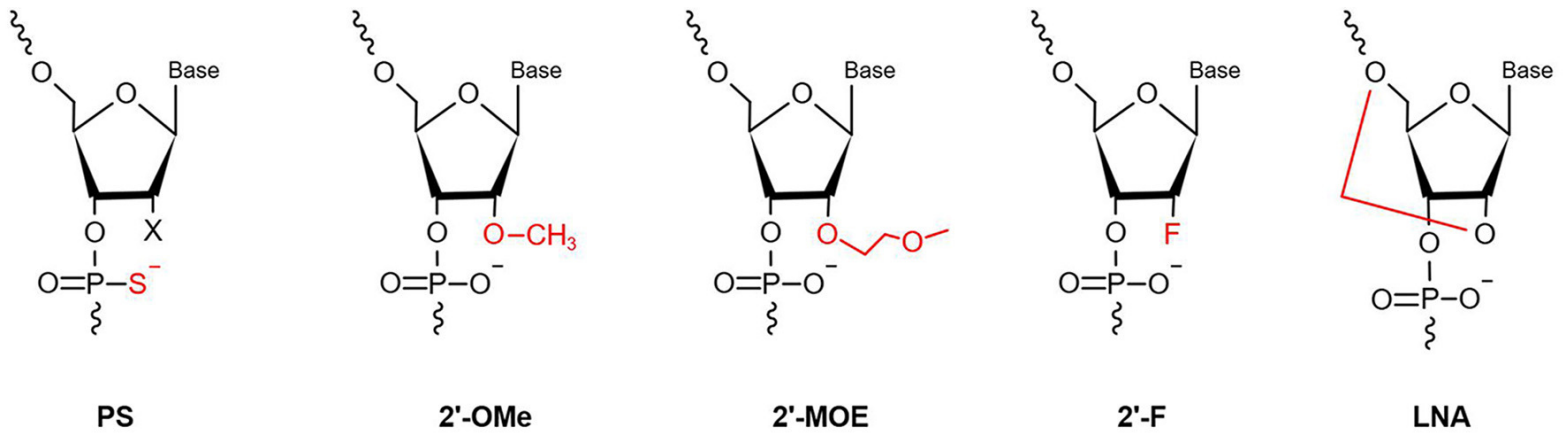
Chemical modifications applied in the AMOs design (PS, phosphorothioate; 2′-OMe, 2′-O-methyl; 2′-F, 2′-fluoro-RNA; 2′-MOE, 2′-O-methoxyethyl; LNA, Locked Nucleic Acid. In red is highlighted the site and the type of modification).

MiRNAs are vulnerable to degradation by nucleases; therefore, chemical modifications to protect miRNAs from nucleases are major solutions that can enhance RNA stability and improve their efficacy (40). Optimal anti-miRNA oligonucleotides (AMOs, or anti-miRs) are designed to be completely complementary with specific mature miRNAs and are chemically modified. Lennox et al. (41) found that chemical modifications of anti-miRs could accelerate their invasion of RISC. At present, the main chemical modification methods used in preclinical studies are phosphorothioate (PS), locked nucleic acid (LNA), and ribose-2'-OH modification (Figure 2).

PS modification, also referred to as oligonucleotide backbone modification, involves replacement of a non-bridging oxygen atom in the phosphodiester bond with a sulfur atom (42). PS can reduce exonuclease- and endonuclease-mediated miRNA degradation; therefore, PS bonds usually are designed near the 5′ and 3′ ends of anti-miRs, to enhance their stability (43). As well as resistance to nucleases, PS-modification can reduce plasma clearance of anti-miRs, increase their stability in serum, and lengthen their half-life, to improve their pharmacokinetic properties, by giving them high affinity with serum albumin (44). Every PS-antimiR substitution reduces the melting temperature of the heteroduplex by 0.5°C (45); however, although the thermal stability of PS-antimiRs is reduced, their increased hybridization specificity and greater nuclease resistance can compensate for this shortcoming. Partial PS oligodeoxynucleotides (ODNs) targeted against the *AT1* receptor mRNA are more effective than full PS ODNs in decreasing blood pressure, as well as having lower cost and improved specificity (46). Compared with other modification strategies, PS modification of anti-miRs is more effective *in vitro*. Two PS modifications of 2'-O-methyl RNA/LNA (2'OMe/LNA) mixtures were reported to be the most efficient of all chemical compounds tested *in vitro* (43).

LNA, as a modified nucleotide analog, is also termed inaccessible RNA, and uses one of its methylene bridges to fix the ribose ring, by linking an oxygen atom at the 2′ position to a carbon atom at the 4′ position. The methylene bridge is usually in the C3′-endo conformation (47). LNA modifications reduce nuclease degradation by increasing RNA stability and avoid off-target effects through 5′ end modification, which prevents molecules from merging into RISC (48, 49). The high affinity of LNA allows shorter anti-miR sequences to be designed for applications, with similar efficiency (50). Obad et al. (50) first demonstrated that it is feasible for tiny LNAs (including those of only 8 nucleotides) to silence miRNAs with negligible off-target effects. Subsequently, Bernardo et al. (51) showed that the seed-targeting 8-mer LNA-modified antimiR-34 (LNA-antimiR-34) could silence the *miR-34* family to prevent pathological cardiac remodeling and improve cardiac function. Another study found that silencing of *miR-34a* using LNA-antimiR-34a in mice with moderate cardiac pathology reduced atrial dilatation and prevented failure of cardiac function (52). A recent study also found that LNA-antimiR-34 doubled the cardiac progenitor growth rate by inhibiting *miR-34* expression (53). The first identified miRNA, *miR-1*, has a critical role in cardiac development and disease (54); it can significantly inhibit the expression of PKCe and HSP60 to promote cardiac injury. Pan et al. (55) verified that LNA modified antimiR-1 (LNA-antimiR-1), at a dose of 1 mg/kg, down-regulated *miR-1* expression by 83% and efficiently reduced cardiac ischemia/reperfusion injury. Wang et al. (56) found that LNA-antimiR-1 remarkably inhibits the production of reactive oxygen species in neonatal Wistar rat cardiomyocytes.

Ribose-2′-OH modification describes the substitution of alternative chemical groups in the 2′ position, primarily 2′-O-methyl (2′-O-Me), 2′-meth-oxyethyl (2′-O-MOE), and 2′-fluoro (2′-F) (57). Ribose-2′-OH modification can enhance nuclease resistance and reduce immunoreactivity (58); however, in most cases, modification is conducted together with LNA or PS modification, or occasionally even both together. Intravenous administration of 2′-O-Me-anti-miRs targeting *miR-16, miR-122, miR-192*, and *miR-194* could decrease the corresponding miRNA levels in mice (59). Similarly, inhibition of *miR-133* using an anti-miR with 2′-O-Me modification leads to hypertrophy *in vivo* (60).

All of the chemical modifications described above have the common advantage of improving nuclease resistance, while PS modification alone also reduces thermal stability and affinity. LNA has become the most commonly used anti-miR chemical modification; however, use of LNA modification alone may decrease RNase H activity. Most anti-miRs tend to be designed with these three chemical modifications in the first and last five nucleotides, referred to as gapmers. These molecules have a good affinity for their target RNAs, effectively improve RNase H activity, and are widely used in many miRNA therapeutics (61, 62).

Overall, AMOs are an effective means of functionalizing miRNAs, both *in vivo* and *in vitro*. The differential effects of chemical modifications of AMOs are summarized in Table 1.

**Table 1:** Advantages and disadvantages of chemical modifications applied to the AMOs.

**Type of modification**	**Advantages**	**Disadvantages**	**References**
PS	•High nuclease resistance •Promoted protein binding •Delaying plasma clearance	•Poor affinity to target RNA •Poor thermal stability	(63–67)
2′-OMe	•Improved affinity to target RNA •Increased thermal stability •No liver toxicity	•Poor stability: compared with 2′-MOE and 2′-F	(63, 65–69)
2′-MOE	•Improved affinity to target RNA •Increased thermal stability •No liver toxicity	•Lower potency: compared with 2′-OMe and 2′-F	(43, 63, 65–67, 70)
2′-F	•Improved affinity to target RNA •Increased thermal stability •No liver toxicity	•No resistance to exonucleases	(63, 65, 66, 71)
LNA	•Improved affinity to target RNA •Increased thermal stability •High potency and specificity •Increased nuclease resistance	•Might present low toxicity (*in vivo*) •Might lowering potency	(41, 50, 63, 65, 72–78)

## Design of MiRNA Delivery Vehicles

When miRNA therapies are applied for heart disease, it is necessary to consider the non-polar and hydrophobic properties of the myocardial cytomembrane, which represent an enormous challenge to miRNA delivery, as miRNAs have a negative charge and are hydrophilic. Therefore, crossing the myocardial cytomembrane is a vital step in transferring miRNAs to their targets successfully and generating effective miRNA therapies. Effective miRNA delivery requires the molecules to exhibit hypocytotoxicity, high transfection efficiency, and specificity. MiRNA delivery vehicles are mainly divided into viral and non-viral vector-based approaches. In this review, we discuss viral-vector-based delivery systems; see refs (79–82) for detailed information on non-viral miRNA vectors. Viral vector delivery aims to reach the target cell using the genome of the virus itself, and has been used extensively for delivery of miRNA therapies. The main viruses used for this purpose are adenoviruses, adeno-associated viruses (AAVs), and lentiviruses.

Adenoviruses, are double-stranded DNA viruses commonly used as viral vectors. As early as 1992, Stratford-Perricaudet et al. (83) found that injecting adenovirus LacZ vectors into neonatal mice resulted in extensive gene transfer in cardiomyocytes. Subsequent studies have shown that adenovirus-mediated *miRNA-24* upregulation can enhance myocardial angiogenesis and blood perfusion in myocardial infarction (MI) tissue. While this approach can induce cardiomyocyte and fibroblast apoptosis, overall, it produced a good MI treatment effect (84). Another study using miRNA therapy for heart failure demonstrated that adenovirus vector contributed to up-regulation of AMPKα2, the direct target of *miR-195a-3p*, helping to relieve cardiac hypertrophy and avoid heart failure (85). Nevertheless, serious flaws have emerged on wide application of adenovirus delivery in clinical studies; for example, use of adenovirus vectors for treatment of malignant intracranial tumors resulted in serious side effects, including headache, change of mental status, and relapsing seizures (86) and such side effects limit the use of adenoviruses for delivery of miRNA therapies.

AAVs are single-stranded, non-enveloped DNA viruses belonging to the “Parvoviridae” family, and are among the most promising vector delivery systems. Unlike other viruses, AAVs are not pathogenic to humans, have minimal immunogenicity, and are low molecular weight (83). AAV2, AAV6, and AAV9 are the most cardiogenic AAVs among AAV1 to AAV9, with AAV6 vector exhibiting the most efficient transduction (87). Bian et al. (88) found that AAV6-mediated over-expression of *miR-199a* promoted the proliferation of human induced-pluripotent stem cells (hiPSC-CMs), which could improve cardiac function and fibrosis. Further, a recent study demonstrated that myocardial tissue in aorta-constricted mice transfected with *miR-124* via AAV9 inhibited the influence of shikonin on sympathetic remodeling, revealing the mechanism underlying the use of shikonin for treatment of chronic heart failure (89). AAV6 and AAV9 have become the main AAVs serotypes used for heart disease therapy. In addition, Gao et al. found that delivery of *miR-19a/19b* using AAV vectors may reduce the cardiac trauma induced by myocardial infarction and protect heart function (90).

A number of studies have reported the use of AAV vectors for miRNA therapy in heart disease. The latest research showed that varying levels of cardiac *miRNA-122* have a crucial effect on the treatment efficiency of AAV vectors regulated by miRNAs (91); however, another report noted severe cardiotoxicity in AAV6 vector-mediated shRNA cardiac gene transfer (92). Notably, AAV antibodies are present in most people, which may influence the effectiveness of vector entry and transgenic expression.

Lentiviruses are a subgroup of retroviruses, and lentivirus vectors are often used for miRNA delivery into myocardial cells. Yang et al. (93) demonstrated that downregulation of *miR-322* via lentiviral transduction could further prevent cardiomyocyte apoptosis induced by hypoxia. Similarly, Wang et al. (94) showed that reduction of *miR-137* levels, mediated by lentivirus vectors, decreased cardiomyocyte apoptosis. Although lentivirus-mediated miRNA therapy has been applied for the treatment of heart disease, the disadvantages of insertional mutagenesis may limit its use (95, 96).

MiRNA-related therapy based on viral vectors has certain advantages. For example, it is easy to generate vectors and they exhibit high transduction efficiency. Moreover, the long-term and stable gene expression mediated by these vectors makes them potentially ideal in this context; however, safety issues represent a huge obstacle that requires further research.

## The Role of MiRNAs in Heart Failure

**Figure 3:**
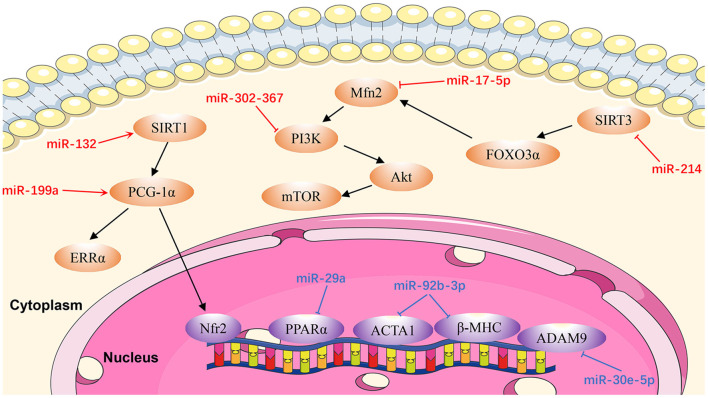
Reviewed miRNAs in cardiac hypertrophic pathways (Red color miRNA, pro-hypertrophic function; Blue color miRNA, anti-hypertrophic function).

**Figure 4:**
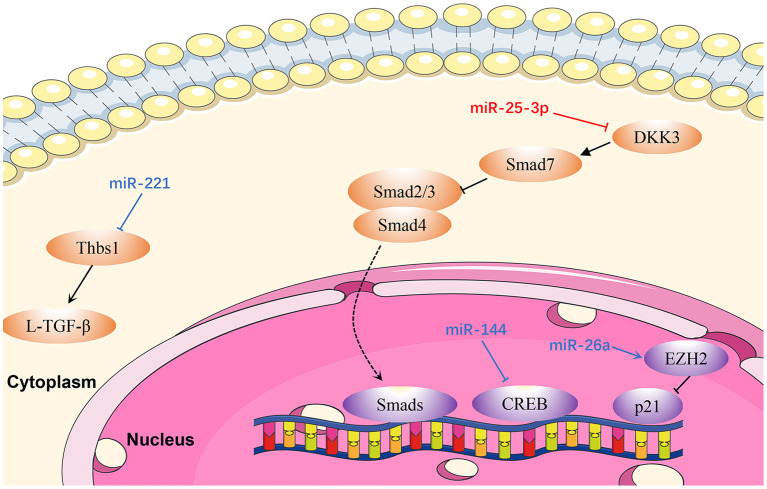
Reviewed miRNAs in cardiac fibrosis pathways (Red color miRNA, pro- fibrosis function; Blue color miRNA, anti- fibrosis function; Dash lines indicate translocation of molecules from cytoplasm to nucleus).

**Figure 5:**
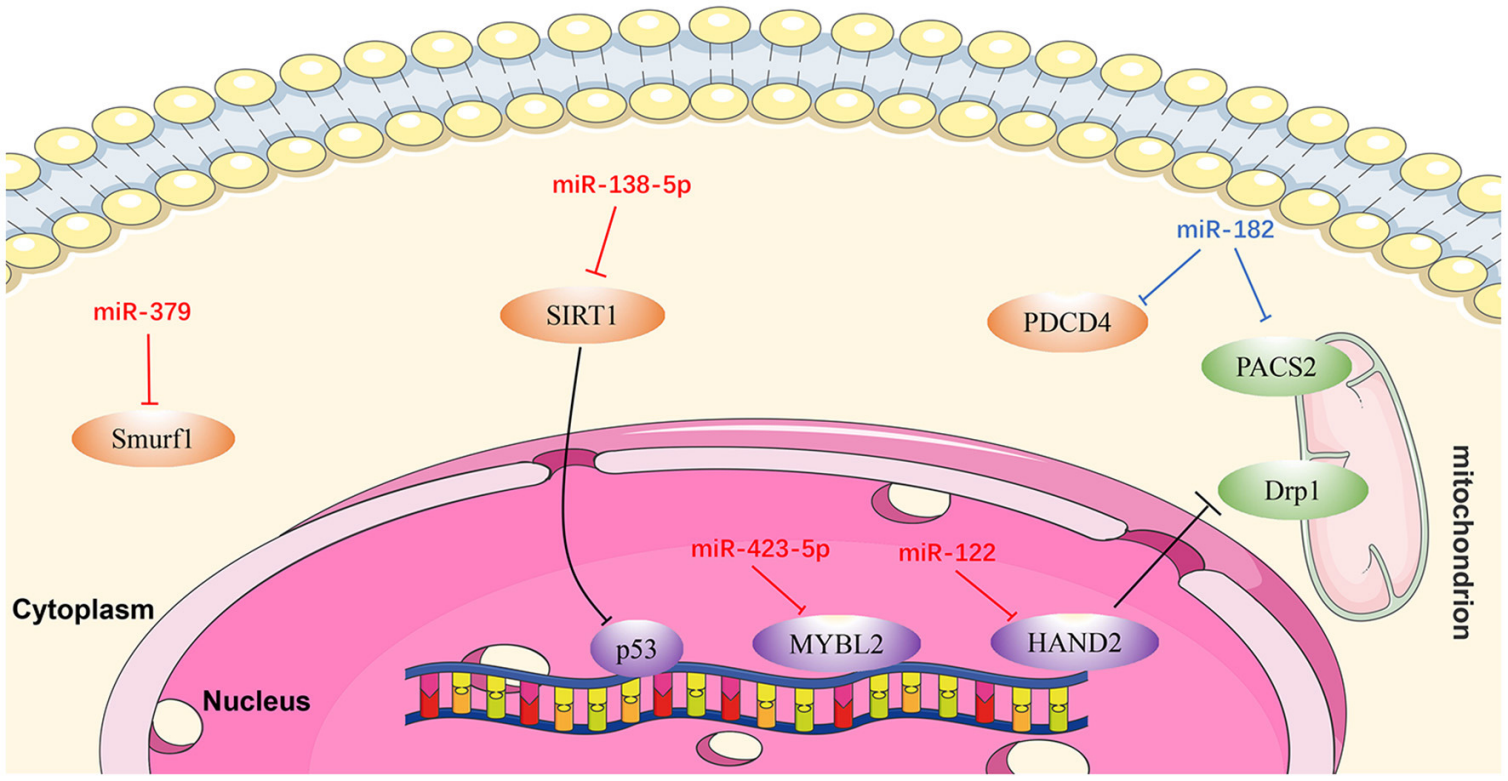
Reviewed miRNAs in cardiomyocyte apoptosis pathways (Red color miRNA, pro-apoptosis function; Blue color miRNA, anti-apoptosis function).

Cardiac remodeling, a basic mechanism underlying HF, is the process of generating changes in the size, shape, and function of the heart, and responds to internal and external cardiovascular injury or risk factors (97). Pathological ventricular remodeling has three main characteristics: extensive fibrosis, pathological cardiomyocyte hypertrophy, and myocardial cell apoptosis (98). In this review, we summarize the regulatory effects of newly-discovered miRNAs in cardiac remodeling, particularly cardiac hypertrophy (Figure 3), fibrosis (Figure 4), and apoptosis (Figure 5).

### MiRNAs in Cardiac Hypertrophy

#### MiR-17-5p

Xu et al. (99) identified *miR-17-5p* as a critical miR that regulates the expression and function of the mitochondrial fusion protein, mitofusin 2 (MFN2). They found that *miR-17-5p* expression in cardiomyocytes was upregulated in rat hearts at 4 weeks after transverse aortic constriction and in an angiotensin II (Ang II)-induced cell hypertrophy model. Mechanistic *in vivo* and *in vitro* studies demonstrated that *miR-17-5p* suppresses autophagy to promote cardiac hypertrophy by inhibiting *Mfn2* expression, and activating the phosphatidylinositol-3-kinase (PI3K)/AKT/mammalian target of rapamycin (mTOR) pathway. Furthermore, Ang II-induced cell hypertrophy in neonatal rat left ventricle myocytes was significantly reversed by an *miR-17-5p* inhibitor. These findings suggest an essential role for *miR-17-5p* in cardiovascular disease and present potential new therapeutic targets in patients with pathological cardiac hypertrophy (99).

#### MiR-29a

*MiR-29a* is significantly associated with cardiac hypertrophy and a potent therapeutic target for the treatment of cardiac hypertrophy (100). Upregulation of *miR-29a in vivo* can attenuate the cardiac hypertrophy response induced by isoproterenol hydrochloride via targeting the nuclear receptor peroxisome proliferator-activated receptor δ (*PPARD*) and downregulating atrial natriuretic factor, suggesting that *miR-29a* can protect the myocardium in cardiac hypertrophy (100).

#### MiR-30e-5p

Upregulation of *miR-30e-5p* has anti-hypertrophic effects in hypertrophic cardiomyocytes induced by Ang II, via targeting a disintegrin and metallopeptidase domain 9 (meltrin gamma) (101). Overexpression of *miR-30e-5p* also attenuates hypoxia-induced apoptosis in an hiPSC-CM injury model by targeting the 3'-UTR of *BCL2L11*, which is an apoptosis activator and autophagy suppressor (102).

#### MiR-92b-3p

According to Yu et al. (103), *miR-92b-3p* is up-regulated in mouse cardiomyocytes under Ang II-induced conditions. *MiR-92b-3p*, is downstream of heart and neural crest derivatives-expressed 2 (*HAND2*), and is involved in cardiac hypertrophy through targeting *ACTA1* and *MYH7*, which are both genes involved in hypertrophy. In Ang II-induced cardiomyocyte hypertrophy, therapeutic studies using *miR-92b-3p* mimics reduced cardiomyocyte cell size and inhibited the expression levels of ACTA1 and MYH7.

#### MiR-132

Another anti-hypertrophic miR, *miR-132*, controls cardiac hypertrophy in a novel porcine model of pressure-overload-induced heart failure via PPARGC1A/NFE2 signaling by targeting SIRT1 (104). Furthermore, Li et al. (105) proved that NFE2 protects the murine heart against pathological cardiac hypertrophy and heart failure. Furthermore, NFE2 is increased in myocardium of anti-miR-132-treated porcine hearts with percutaneous transverse aortic constriction compared with the control group at the 8-week time point, indicating that this could be an effective therapeutic strategy for patients with heart failure (104). Therefore, antimiR-132 may be pivotal in mediation of pathologic heart hypertrophy.

#### MiR-199a

Yan et al. (106) demonstrated that *miR-199a* is increased in pressure-induced cardiac hypertrophy, while inhibition of *miR-199a* mitigates cardiac hypertrophy *in vitro*. Furthermore, anti-miR-199a attenuates cardiac hypertrophy and restores cardiac function *in vivo* through the PPARGC1A (PGC-1α)/ESRRA (ERRα) axis. The mechanism underlying restoration of mitochondrial structure and function in anti-miR-199a-treated mice involves the downstream pathways of mitochondrial fatty acid oxidation and oxidative phosphorylation (107).

#### MiR-214

*MiR-214* levels are dramatically raised in Ang II-infused mice (108). Hypertrophic stimuli cause increased expression of *miR-214* in cardiomyocytes, resulting in *miR-214*-mediated hypertrophic growth. In contrast, inhibition of *miR-214* confers protection from Ang II-mediated hypertrophy *in vivo*. These effects are attributable to increased expression of SIRT3, which participates in cardiomyopathy pathogenesis by inducing mitochondrial injury and energy metabolism disorder. Using a dual-luciferase reporter assay Ding et al. (108) demonstrated that SIRT3 was a direct target of *miR-214*. These results provide information about the biological functions of *miR-214* and indicate that it may be a promising target for cardiac hypertrophy therapy.

#### MiR-302/367

An initial study of the role of *miR-302/367* focused on its function in H9c2 cells treated with Ang II. The expression of *miR-302/367* is up-regulated and leads to autophagy in an *in vitro* hypertrophic model (109). In addition, loss- and gain-of-function assays demonstrated that *miR302/367* aggravates cardiac hypertrophy by inhibiting autophagy. Jin et al. (109) demonstrated that upregulation of *miR302/367* expression acts as an endogenous inhibitor of autophagy in hypertrophic H9c2 cells through PTEN/PI3K/AKT/mTORC1 signaling. Anti-hypertrophic effects were observed in Ang II-induced hypertrophic H9c2 cells via the inhibition of *miR302/367*. These findings reveal the critical role of *miR302/367* in mediating hypertrophy of the heart through PTEN/PI3K/ AKT/mTORC1 signaling (109).

### MiRNAs in Cardiac Fibrosis

#### MiR-25-3p

Zeng et al. (110) proved that *miR-25-3p* expression is mediated by NF-κB signaling in cardiac fibrosis, and the regulation of fibrosis-related gene expression by *miR-25-3p* is observed both *in vitro* and *in vivo*. Mechanistically, DKK3 expression is reduced in cardiac fibrosis under modulation by *miR-25-3p* via inhibition of SMAD7 and promotion of SMAD3 and fibrosis-related gene expression (110). Hence, *miR-25-3p* represents a potentially promising drug target in cardiac fibrosis.

#### MiR-26a

*MiR-26a* is downregulated in the plasma and myocardium of spontaneously hypertensive rats (111). Zhang et al. (111) demonstrated that *miR-26a*-deficient mice exhibit increased myocardial fibrosis, whereas overexpression of *miR-26a* significantly inhibited myocardial fibrosis *in vivo* and Ang II-induced fibrogenesis in cardiac fibroblasts by directly targeting connective tissue growth factor and SMAD4 (111). In addition, cardiac fibroblast proliferation is inhibited by *miR-26a* via the EZH2/P21 pathway. These results reveal a novel role for *miR-26a* in hypertensive myocardial fibrosis and provide a possible treatment strategy for this condition.

#### MiR-144

Li et al. (112) demonstrated that *miR-144* was dramatically down-regulated in response to pathological stimuli. Upregulation of *miR-144* significantly decreased the proliferation and migration ability of cardiac fibroblasts, and also reduced the transformation from fibroblasts to myofibroblasts, whereas downregulation of *miR-144* could reverse these effects (113). In that study, bioinformatics analysis and luciferase reporter assays demonstrated that *miR-144* directly targets and downregulates *CREB* expression in cardiac fibroblasts treated with Ang II (113).

#### MiR-221

Zhou et al. (114) proved that *miR-221* inhibits the activation of L-TGF-β1 by directly targeting THBS1, thus mitigating cardiac fibrosis and improving cardiac function. Similarly, *miR-221* mimics transfected into rat cardiac fibroblasts induced by kidney failure resulted in reduction of cardiac fibrosis. Therefore, *miR-221* mimics are a promising therapeutic target in cardiac fibrosis (114).

### MiRNAs in Cardiomyocyte Apoptosis

#### MiR-122

*MiR-122* is one of several miRNAs elevated in patients with HF, and plays a pivotal role in cardiac insufficiency by inducing cardiomyocyte apoptosis (115). Shi et al. (116) found that DRP1 levels were increased in response to upregulation of *miR-122*, indicating that apoptosis (and cardiac dysfunction) increase through DRP1-mediated up-regulation of mitochondrial fission. Furthermore, *miR-122* interacts directly with a transcription factor regulating its own expression, and the effect of miR-122 on DRP1 is mediated by *HAND2* (116). These findings demonstrate that *miR-122* exerts a regulatory role in cardiomyocyte apoptosis via suppressing *HAND2*, which results in increased expression of *DRP1*, and ultimately leads to apoptosis (116).

#### MiR-138-5p

Recent research showed that *SIRT1* is a potential candidate target gene of *miR-138-5p* and that *miR-138-5p* levels were negatively correlated with those of *SIRT1* in cardiomyocytes (117). SIRT1 alleviates HF by enhancing P53 deacetylation, thereby inhibiting cardiomyocyte apoptosis. *MiR-138-5p* can decrease SIRT1 expression in H_2_O_2_-induced AC-16 and HCM cells by activating P53 signaling. By contrast, *in vitro* knockdown of *miR-138-5p* has a clear protective effect on cardiomyocytes in HF models (118). In summary, *miR-138-5p* inhibits SIRT1 enzyme activity by activating P53 signaling, resulting in deterioration of HF (118).

#### MiR-182

Rapid ventricular pacing downregulates *miR-182* levels and induces cardiomyocyte apoptosis and HF in rats (119). An *in vitro* study illustrated that PDCD4 can promote tumor cell apoptosis by affecting the translation of eukaryotic initiation factor-4A (eIF4A) and eIF4G (120). PACS2 is an initiator of apoptosis, which accelerates displacement of mitochondrial death pathway agonists (121, 122). Expression levels of both *PDCD4* and *PACS2* were inhibited following upregulation of *miRNA-182*, while the apoptotic rate of cardiomyocytes in HF rats decreased. These results suggest that *miR-182* inhibits cardiomyocyte apoptosis induced by non-ischemic HF via downregulating *PDCD4* and *PACS2* (119).

#### MiR-379

Chen et al. (123) discovered that the *KLOTHO* gene is a suppressor of aging whose deficiency can damage heart function and lead to heart failure. In addition, an antimir for *miR-379* can prevent H9c2 cell apoptosis induced by *KLOTHO* deficiency, while *miR-379* mimics can induce apoptosis of H9c2 cells (123). These authors also proved that the inhibition of SMURF1 may be related to H9c2 cell apoptosis induced by *miR-379*. In summary, *miR-379* promotes cardiomyocyte apoptosis via targeting SMURF1, which is essential for *mir-379*-induced apoptosis. Anti-mir-379 represents a potential therapeutic target for cardiomyocyte apoptosis (123).

#### Mir-423-5p

A recent study confirmed that downregulation of *miR-423-5p* reduced hypoxia/reoxygenation-mediated cardiomyocyte injury by targeting *MYBL2* in cardiomyocytes through the WNT/β-catenin signaling pathway, and that this process can be reversed by treatment with an *miR-423-5p* inhibitor (124).

Overall, various miRNAs modulate different mechanisms and signaling pathways that promote or protect against heart failure and those miRNAs are potential therapeutic targets in patients with heart failure.

However, a number of obstacles limit the clinical applicability of anti-miRNA agents as cardiovascular disease therapeutics. In particular, their potential off-target effects, which will result in unwanted toxicity, remain major challenges to be overcome (125). For example, Hinkel et al. (126) demonstrated that the administration of antimiR-21 in a pig model of HF caused considerable downregulation of miR-21 in the lung and kidney, which may cause unwanted side effects in these organs. This finding had also been reported in other investigations and was not unexpected (57). For the treatment of HF, it is necessary to develop a non-invasive and efficient tissue-specific drug delivery method. Moreover, it should be noted that many microRNA's systems effect is complicated by the fact that some microRNAs are protective in cancer but detrimental in cardiovascular setting. For example miR-34 (127, 128). Cardiotoxicity of a microRNA therapeutic is definitely something that limited the full potential of a specific microRNA therapeutics. The underlying mechanisms of a microRNA's effect or just with its seed sequence still need to be elucidated.

Identification of the specific target of an miRNA is one approach for determining the role of an miRNA in biological or pathological processes (129); however, a single miRNA can have thousands of targets. Further, experimentally determined miRNA genes and targets are far removed from clinical and translational demands. At present, machine learning-based computational targets and gene predictions have become an intense focus in this field (130), and have the potential to initiate a new era in miRNA research in various diseases (131). A summary of all miRNAs known to be involved in human cardiovascular diseases and their predicted target genes is presented in Table 2.

**Table 2:** All miRNAs summarized in this review and their predicted target genes in human cardiovascular diseases (TargetScanHuman Release 7.2).

**MiRNA**	**Predicted target genes**
MiR-17-5p	*BVES MKL2 HEG1*
MiR-29a	*HAND2 HSPB7 CASQ2 ATP2A2 ANKRD1 MYOCD MKL2 MEF2A*
MiR-30e-5p	*ACTC1 CLCF1 ATP2A2 MKL2 MEF2D*
MiR-92b-3p	*ACTC1 ATP2A2 HAND1 HAND2 HEG1 MEF2D*
MiR-132	*MEF2A*
MiR-199a	*MEF2C MKL2 MEF2D*
MiR-214	*MEF2A MKL2 MEF2C MEF2D*
MiR-302/367	*LBH HEG1 MKL2 ANKRD1 BVES ATP5A1 ACTC1 MEF2A* *MEF2C MEF2D HAND2 MYZAP MYOCD ATP2A2*
MiR-25-3p	*HAND1 HAND2 HEG1 MEF2D ACTC1*
MiR-26a	*MKL2 ACTC1 MEF2A MEF2C MYOC*
MiR-144	*HEG1 MKL2 MEF2A MEF2C MEF2D*
MiR-221	*HEG1 MYZAP MYOCD*
MiR-122	*MEF2C MEF2D ATP5A1*
MiR-138-5p	*BVES*
MiR-182	*MKL2 MEF2A MEF2C MEF2D MYOCD*
MiR-379	*MEF2D MGST1*
Mir-423-5p	*MYBL2*

## A Milestone Breakthrough in the Treatment of Heart Failure: First-in-human Evidence (CDR132L)

Progress over the past three decades has significantly improved the development of nucleic acid therapeutics. The first-in-human study of an miRNA-based therapy was MRX34, a liposome-based *miR-34a* mimic, for the treatment of advanced solid tumors in April 2013 (132, 133). This provided valuable insights into the potential for application of new oligonucleotide-based drugs in oncology (134). To date, several RNA-targeted drugs have been approved for commercial use, while others are in the final phases of clinical trials (135–137).

*MiRNA-132-3p* (*miR-132*) is a non-coding RNA whose cardiac expression is up-regulated in patients under cardiomyocyte stress. High expression of *miR-132* in heart tissue leads to progressive cardiac remodeling, and thereby HF events. In addition, preclinical animal experiments show that *miR-132* can affect signaling pathways related to cardiomyocyte growth, autophagy, calcium handling and contraction and, more significantly, can down-regulate FOXO3 levels and inhibit the expression of genes related to intracellular calcium handling and contraction, which can lead to cardiac remodeling (138). Hence, *miR-132* has attracted the attention of clinicians as a promising molecular target for HF treatment.

CDR132L, a synthetic lead-optimized oligonucleotide targeting *miR-132*, was the first *miRNA-132* inhibitor. In related preclinical studies, the application of CDR132L significantly improved cardiac function, and can attenuate, or even reverse, HF (139).

Based on these findings, Täubel et al. published the first in-human clinical trial of CDR132L in patients with HF. The study was a randomized, double-blind, placebo-controlled, dose-escalation clinical trial. This Phase 1b clinical trial confirmed for the first time that CDR132L is safe and well-tolerated in humans, with no obvious toxicity. Simultaneously, CDR132L can significantly reduce the level of NT-proBNP, a biomarker of heart failure and narrow QRS complex, and lead to positive trends in myocardial fibrosis markers (140). This represents a huge transformation in treatment for patients with heart disease, from symptomatic and supportive therapies, to etiological treatments (141). As clinical researchers and medical workers, while maintaining a cautious attitude toward data generated from small sample sizes, we should also actively recognize the “encouraging” pharmacodynamic performance of CDR132L. Larger-scale clinical studies are needed in the future to further confirm the positive role of CDR132L in the treatment of heart failure. More broadly, this research can inspire more studies investigating RNA-based therapeutics for cardiovascular disease.

## Conclusion

As miRNAs can target a wide range of genes (mRNAs), and an individual gene can be regulated by multiple miRNAs, the complex communication networks between these two molecule types indicate that miRNAs can regulate numerous different biological phenomena, ranging from hypertrophy and fibrosis to angiogenesis, among other processes. Therefore, miRNAs have significant potential for preventing, alleviating, and even restoring cardiac dysfunction, such as adverse ventricular remodeling. Further, therapeutic cocktails including miRNA inhibitors and treatment strategies targeting multiple genes involved in disease development may produce remarkable results; however, the broad clinical application of miRNA-related therapy must first overcome several obstacles, including targeted delivery, off-target effects, and hepatic/renal toxicity. Thus, further attempts to promote clinical application of miRNA-related therapeutics are urgently required.

## Author Contributions

HZ drafted the manuscript. CF designed the study. WT, JY, JP, and JG revised the manuscript. HZ and WT were responsible for the collection of data or analysis. All authors read and approved the final manuscript.

## Funding

This work was supported by the Key Project of Science and Technology of Hunan Province (No. 2020SK53420 to JY). Hunan Province Outstanding Postdoctoral Innovative Talent Project (2021RC2106 to CF).

## Conflict of Interest

JG and CF was employed by the company, Hunan Fangsheng Pharmaceutical Co. Ltd. The remaining authors declare that the research was conducted in the absence of any commercial or financial relationships that could be construed as a potential conflict of interest.

## Publisher's Note

All claims expressed in this article are solely those of the authors and do not necessarily represent those of their affiliated organizations, or those of the publisher, the editors and the reviewers. Any product that may be evaluated in this article, or claim that may be made by its manufacturer, is not guaranteed or endorsed by the publisher.

## Abbreviations

AAVs: adeno-associated viruses
AMOs: anti-miRNA oligonucleotides
Ang II: angiotensin II
HF: Heart failure
hiPSC-CMs: human induced-pluripotent stem cells
iCMs: induced cardiomyocytes
LNA: locked nucleic acid
MI: myocardial infarction
miRNAs: microRNAs
mRNAs: messenger RNAs
mTOR: mammalian target of rapamycin
ODNs: oligodeoxynucleotides
PS: phosphorothioate
PI3K: phosphatidylinositol-3-kinase
UTR: untranslated region.
